# Critical periods after stroke study: translating animal stroke recovery experiments into a clinical trial

**DOI:** 10.3389/fnhum.2015.00231

**Published:** 2015-04-29

**Authors:** Alexander W. Dromerick, Matthew A. Edwardson, Dorothy F. Edwards, Margot L. Giannetti, Jessica Barth, Kathaleen P. Brady, Evan Chan, Ming T. Tan, Irfan Tamboli, Ruth Chia, Michael Orquiza, Robert M. Padilla, Amrita K. Cheema, Mark E. Mapstone, Massimo S. Fiandaca, Howard J. Federoff, Elissa L. Newport

**Affiliations:** ^1^Department of Rehabilitation Medicine, Center for Brain Plasticity and Recovery, Georgetown University and MedStar National Rehabilitation HospitalWashington, DC, USA; ^2^Department of Neurology, Georgetown UniversityWashington, DC, USA; ^3^Department of Kinesiology and Occupational Therapy, University of WisconsinMadison, WI, USA; ^4^Department of Biostatistics, Georgetown UniversityWashington, DC, USA; ^5^Department of Neuroscience, Georgetown UniversityWashington, DC, USA; ^6^Departments of Oncology and Biochemistry, Georgetown UniversityWashington, DC, USA; ^7^Department of Neurology, University of RochesterRochester, NY, USA

**Keywords:** stroke rehabilitation, cerebrovascular disorders, critical period, motor recovery, multi-omics, adaptive randomization

## Abstract

**Introduction:** Seven hundred ninety-five thousand Americans will have a stroke this year, and half will have a chronic hemiparesis. Substantial animal literature suggests that the mammalian brain has much potential to recover from acute injury using mechanisms of neuroplasticity, and that these mechanisms can be accessed using training paradigms and neurotransmitter manipulation. However, most of these findings have not been tested or confirmed in the rehabilitation setting, in large part because of the challenges in translating a conceptually straightforward laboratory experiment into a meaningful and rigorous clinical trial in humans. Through presentation of methods for a Phase II trial, we discuss these issues and describe our approach.

**Methods:** In rodents there is compelling evidence for *timing effects in rehabilitation*; motor training delivered at certain times after stroke may be more effective than the same training delivered earlier or later, suggesting that there is a critical or sensitive period for strongest rehabilitation training effects. If analogous critical/sensitive periods can be identified after human stroke, then existing clinical resources can be better utilized to promote recovery. The Critical Periods after Stroke Study (CPASS) is a phase II randomized, controlled trial designed to explore whether such a sensitive period exists. We will randomize 64 persons to receive an additional 20 h of upper extremity therapy either immediately upon rehab admission, 2–3 months after stroke onset, 6 months after onset, or to an observation-only control group. The primary outcome measure will be the Action Research Arm Test (ARAT) at 1 year. Blood will be drawn at up to 3 time points for later biomarker studies.

**Conclusion:** CPASS is an example of the translation of rodent motor recovery experiments into the clinical setting; data obtained from this single site randomized controlled trial will be used to finalize the design of a Phase III trial.

## Background

Using animal models of stroke, substantial scientific progress has been made in the understanding of the neural substrates of recovery after brain injury. Experimental studies of motor training after injury show that motor function can be improved significantly when a number of recovery and training variables are controlled. The experiment of Biernaskie et al. (2004) has been particularly intriguing given the finding of a sensitive period after experimental stroke in which rodents are most responsive to motor training in a specific time window soon after stroke. This finding has provoked much discussion in the stoke rehabilitation research community, since of course one wants to rehabilitate stroke patients at the time after stroke when therapies can be most effective. In this paper, we discuss the challenges faced by clinical trialists in translating a conceptually straightforward rodent experiment into a stroke rehabilitation clinical trial. We present our methods for the Critical Periods after Stroke Study (CPASS) as one example of the choices that can be made in testing whether promising findings in rodents have relevance in rehabilitation of patients with stroke.

The CPASS trial is designed to translate important findings from the rodent motor recovery literature into the human clinical trial setting. Adapting the critical elements of the rodent studies to the stroke rehabilitation setting requires a series of decisions and accommodations. In this paper, we review and discuss these considerations and how we have addressed them. Where possible we have retained essential elements of the rodent studies, including manipulation of intervention timing, randomization, standardized motor training paradigm based on a highly salient reward, and the use of motor performance measures. Data obtained from this randomized controlled trial will be used to formulate more effective treatments to better focus on the needs of individuals with stroke.

### Approaching the translation of animal experiments into clinical trials

Table 1 displays the many advantages of rodent experiments; these advantages allow exacting study of the biology of mammalian brain recovery, and the most unequivocal demonstration of the impact of putative motor training interventions. The rodents can be healthy young animals predictably available through breeding or purchase, eliminating confounds of differences in rearing, medical conditions and post-injury mortality. The ability to test a group of subjects simultaneously eliminates any drift in study or training procedures. Heterogeneity across animal subjects can be limited by the use of a single gender and a genetically homogenous strain. Brain lesions can be standardized and made in a brain that is otherwise pristine. Motor training protocols can be uniform and timed exactly. Food can be used as a highly motivating reward, and subjects are not lost to follow-up. Biological mechanisms can be studied using tissue and molecular techniques requiring sacrifice of the animals.

**Table 1:** **Issues in translation from rodent experiments to human clinical trials**.

**Desirable characteristic of rodent experiment controlled by investigator**	**Limitations in translation to human subjects**	**Methods to mitigate human subjects limitations**
**EXPERIMENTAL SUBJECT POPULATION**		
Genetic background Rearing conditions Subject availability Age, gender medical comorbidities Inexpensive to obtain Relatively distant oversight by institutional review board and animal care committee	Most human populations genetically heterogeneous Wide variety of socioeconomic and activity backgrounds Enrollment dependent on flow of stroke patients, and lengthy periods are usually required to identify and enroll sufficient numbers of participants. Close review and oversight by Institutional Review Boards; heavy documentation required	Measure important personal characteristics, analyze as covariates Use randomization method to balance for important subject-specific covariates Adjust inclusion/exclusion criteria to minimize heterogeneity without unduly affecting enrollment Adaptive trial designs to ensure participants are randomized only to promising study arms Do studies in large centers with high patient throughput, or multicenter trials
**BRAIN LESIONS**		
Defined time of onset Reproducible mechanism of injury Reproducible lesion location and size Injuries occur in otherwise pristine brain Muted immunological response to injury Quick and relatively complete motor recovery; greater recruitment of brainstem and extrapyramidal structures	Time of onset can be ambiguous Multiple stroke mechanisms Cause of stroke often undetermined Wide variety of stroke lesions Prior stroke and white matter changes present Greater and more variable immunological response to injury Slower and variable motor recovery	Use study designs that do not require precise time of onset (e.g., wide enrollment windows) Use randomization method to balance for important subject-specific covariates Use of stratification in design Use study designs that do not require specific stroke mechanisms, or ignore lesions and recruit based on clinical impairments Require specific lesions or lack of background brain changes
**TRAINING CONDITIONS**		
Timing Amount Consistency of training paradigm Motivation: High with food rewards	Little control over timing of patient presentation acutely, during inpatient or outpatient rehab, or chronic care setting Need to adapt training to clinical environment, which cannot be controlled by research team Need to recruit over months or years can lead to drift in participant training and assessment methods Amount of training can be dictated by unrelated factors (insurance, transportation, etc) Training is an interaction between unique therapist and unique participant with specific impairments, thus difficult to standardize Motivation to participate in training program limited by lack of knowledge about stroke recovery, cognitive impairment, depression	Focus study activities at specific clinical milestones (rehabilitation admission, initiation of outpatient therapies, etc.) Study provides pragmatic support to overcome insurance payment and transportation barriers Treatment protocols that are flexible for participant needs, but reproducible and well quantified Scheduled audits of training protocol execution and outcome assessment Access increased motivation through patient-centered training activities
**BACKGROUND CONDITIONS**		
Post-injury environment Follow up Diet and activity Motor training outside of study	Differences in care across multiple institutions Variations in home environment and social support Loss to follow up due to subject withdrawal, moving away, medical events Little control over diet Little control over therapies prescribed outside of study	Recruit from one large institution or standardize practices across multiple sites Measure home environment and social support, treat as covariate Select participants who are socially stable and unlikely to withdraw or move Discourage or prohibit outside therapies as a condition of study participation; or measure and treat as a covariate
**ABILITY TO STUDY MECHANISM OF RECOVERY**		
Sacrifice of subjects for anatomic and metabolic studies Homogenous genetic background	Brain tissue rarely available Cerebrospinal fluid difficult to obtain Only non-invasive or minimally invasive assessments available	Use of inferential evaluations: multi-omics of peripheral blood, functional MRI, electrophysiology

Designing a human stroke motor recovery trial tightly linked to the methods used in rodent motor recovery experiments involves a series of adaptations. These adaptations attempt to minimize the real-world limitations of clinical research and to maximize the clinical and scientific utility. The middle column of Table 1 displays some of the challenges faced by clinical trialists as they adapt these experiments to the clinical setting. A simple direct translation of rodent methods into humans can result in a trial that would be straightforward to design, but impractical to execute. For example, an investigator may want to insist that a single, specific lesion type be present for an individual to enroll in a clinical trial. This insistence might be scientifically justifiable, but impossible to execute because of the difficulty of finding sufficient numbers of individuals who suffered the needed infarct, meet other inclusion criteria, and are willing and able to participate in a trial. Similarly, challenges exist in enforcing exact timing of treatments, the content of treatments, obtaining motivated participation in training, and simply locating the individual to collect outcome measures. Approaching the biology of brain recovery in humans is also more challenging because of the infeasibility of recovering brain tissue; even lumbar punctures limit large scale participation in trials.

Clinical trial methods can mitigate the limitations of the stroke rehabilitation clinical setting; many examples are listed in the third column of Table 1. For example, the problem of identifying large numbers of participants can be limited through the use of adaptive trial designs, ensuring that participants will be randomized only to study arms that are promising. Less stringent inclusion/exclusion criteria can increase participant accrual, and the accompanying increase in heterogeneity across subjects can be managed using adaptive randomization strategies to minimize differences between study groups. Treatments that begin on a single preselected day in animals are not realistic in the fluid and unpredictable clinical setting, but can be replaced by treatment initiation intervals, allowing flexibility to the participant and research team. In other cases, investigators must simply make choices based on knowledge of the population, clinical setting, or treatment techniques. The number of choices can be quite large, and often the importance of individual choices is visible only in retrospect at the end of an expensive multiyear effort to answer what to all initial appearances is a straightforward question.

### Laboratory-based work in critical periods after stroke

In current practice, as it becomes possible for the patient to participate after stroke, rehabilitation begins. This rehabilitation is initially superimposed on a background of resolving brain edema, inflammation and apoptosis, which are not thought to be materially influenced by experiences such as motor training (Carmichael, 2006; Cramer, 2008).

In contrast, rehabilitation itself is a mixture of compensation and learning. New learning, particularly that obtained via activity-based therapies (ABT's) (Dromerick et al., 2006), is thought to be accomplished by experience driven neuroplasticity (Kleim and Jones, 2008; Carter et al., 2010). The patient relearns prior methods of accomplishing everyday tasks and when necessary, learns new ways to accomplish goals through a combination of newly acquired compensatory strategies (Nakayama et al., 1994) and restoration of motor, sensory, and cognitive function in uninjured tissues (Lum et al., 2004, 2009; Levin et al., 2009). Since these processes often do not return the patient back to pre-stroke levels of function, understanding and exploiting animal findings of critical or sensitive periods in rehabilitation is an important approach to improving treatment.

These putative periods of greatest responsiveness after stroke have been hypothesized to be analogous to the “critical periods” in normal development (Murphy and Corbett, 2009). In the developing brain, critical periods are defined as times of greatest sensitivity to exogenous influences or experiences. Critical periods for the effect of experience on the formation of neural circuits and on the behaviors they control have been demonstrated, for example, in the establishment of ocular dominance columns and stereopsis in the visual system (Hubel and Wiesel, 1970), in the formation of attachment and species identification in a variety of avian species (Hess, 1973), and in vocal learning in songbirds (Marler, 1970) and in humans (Johnson and Newport, 1989; Newport, 1990). The molecular mechanisms underlying the opening and closing of developmental critical periods are beginning to be well understood (Hensch, 2005), and there are now even examples of “reopening” early critical periods during adulthood (Bavelier et al., 2010; Zhou et al., 2011).

The work of Biernaskie et al. (2004) suggests that certain periods after stroke may constitute a period of enhanced plasticity, analogous to a critical period during which the recovering brain is most sensitive to exogenous stimuli and experience. Thus, there may be an optimal time when stroke patients might show the largest improvement from therapy; and, should stroke patients not receive this optimally timed therapy, it is possible that the opportunity for optimal recovery could be irrevocably lost. Given the discontinuities in US health care, it is common for patients' therapy to be delayed for personal, medical or insurance reasons (Ostwald et al., 2009); even inpatient rehabilitation admission does not guarantee substantial amounts of motor training (Lang et al., 2009). Carefully executed studies demonstrating the optimal timing of therapies will help clinicians and policymakers ensure delivery of effective rehabilitation.

Most of the evidence regarding the timing effects of post-stroke motor training focuses on the behavioral, cellular, and molecular mechanisms of neuroplasticity. More recently, animal models demonstrate that genes involved in normal development (and that are quiescent in adulthood) are expressed at high levels in the first weeks after stroke and then decline, with distinct temporal patterns of gene expression after injury (Carmichael, 2003, 2006). This pattern of gene expression is consistent with the notion of an injury-induced recapitulation of development-like processes which occur during a period of enhanced plasticity. Most of these findings focus on the first weeks after stroke; our study design has three relevant time points (early/acute, subacute, and chronic), in order to best assess and locate such an effect, if indeed it occurs in human patients.

There are two major findings regarding treatment timing in animal models of stroke. First is the work of Schallert (Kozlowski et al., 1996; Humm et al., 1998) and others (Bland et al., 2000) showing that very early and intensive training can reduce recovery after experimental stroke and enlarge lesions. This may have been confirmed in humans in our own work (Dromerick et al., 2009), when we found that very intense motor training early after stroke led to worse outcomes. Second, and more optimistic is the work of Biernaskie et al. (2004) where the question of timing effects was directly addressed. They randomized lesioned animals to receive focused motor training at 5, 14, or 30 days after lesioning. They found that the best response to training started at 5 days after lesioning; an intermediate response was present when training was initiated at 14 days; and therapy beginning at 30 days resulted in the same motor outcome as controls who were not trained at all. This powerful pattern of results suggests that critical periods in stroke recovery do exist in adult mammals (Murphy and Corbett, 2009).

### Human and clinical data regarding timing effects in rehabilitation treatment

Whether and how the results of Biernaskie et al. translate to human stroke patients is unknown. Few prospective human studies directly address optimal timing of rehabilitation. Natural history studies show that recovery after stroke in humans is fastest in the first weeks (Wade and Hewer, 1987; Jorgensen et al., 1995a,b); this period coincides with both the onset of rehabilitation treatment and the time that homeostasis is re-established, as described above. Clinicians have written for decades regarding the features of motor recovery that seem to resemble patterns of normal motor development (Cramer and Chopp, 2000; Pollock et al., 2007; Kollen et al., 2009). Retrospective data from clinical populations suggest that early initiation of rehabilitation is associated with better outcome (Wylie, 1970; Feigenson et al., 1977; Kotila et al., 1984; Rossi et al., 1997). However, these studies are confounded because patients who present late to rehabilitation are generally sicker and more severely affected, and thus less likely to improve regardless of timing of care (Ween et al., 1996). Some, but not all (Gagnon et al., 2006) newer studies using case control methods (Paolucci et al., 2000) or large multicenter cohorts (Maulden et al., 2005) have also found better responses early.

Secondary analyses of existing clinical trials are mixed. The EXCITE trial (Wolf et al., 2006) evaluated whether constraint therapy was superior to an uncharacterized “usual and customary care” (UCC) control in improving UE motor impairment; secondary analyses suggested that the participants treated earlier had a better motor outcome than those treated later (Wolf et al., 2010). The LEAPS trial (Duncan et al., 2011) of body-weight supported treadmill training for gait did not confirm a timing effect. LEAPS found that there were persistent treatment responses at both time points tested (2 and 6 months), but there were no significant outcome differences between the earlier and later groups. VECTORS, a single center Phase II trial (Dromerick et al., 2009) of constraint therapy early after stroke addressed dosing and therapy content rather than timing, but the results at this earlier time period suggested an inverse dose response relationship (at high doses, more therapy led to less motor recovery). A more recent study testing additional rehabilitation therapy early after stroke did not confirm this inverse dose phenomenon and suggested greater ipsilesional cortical activation on functional MRI in those randomized to extra therapy (Hubbard et al., 2014). A recent trial in ICH patients suggested a possible mortality benefit with early therapy (Liu et al., 2014). Preliminary data from AVERT (Bernhardt et al., 2008), an early mobilization RCT, are promising but enrollment is still ongoing.

## Study rationales and hypotheses

The overall goal for the CPASS trial is to identify a critical period after stroke in which patients are particularly responsive to motor training interventions. We hope simply to elicit a signal that a critical period exists; optimization of dosing or treatment strategy would come in subsequent studies.

**Figure 1:**
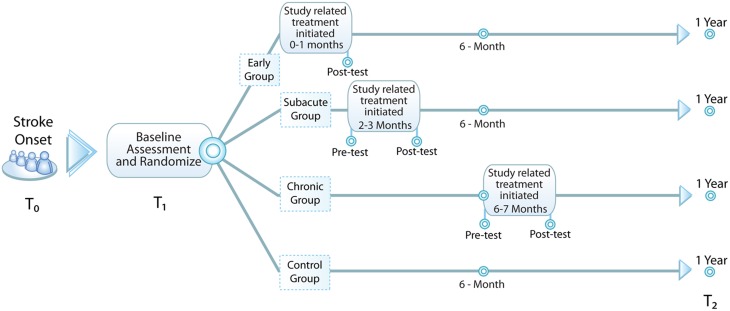
**Study design**. Baseline assessment (T_1_) occurs within the first month following stroke and subjects are randomized to one of four groups: early—additional 20 h of occupational therapy (OT) initiated <1 mo. from stroke onset, subacute—additional 20 h of OT initiated 2-3 mo. from stroke onset, chronic—additional 20 h of OT initiated 6-7 mo. from stroke onset, and control—no additional OT. All subjects are reassessed at 1 year (T_2_) to evaluate for durable change in study-related outcome measures.

Our approach is to use a bolus of standardized motor therapy to elicit a motor improvement during a specific time period indicative of a critical period. Our hypothesis for the CPASS Phase II trial is that, compared to individuals randomized to the control condition or to the subacute (2–3 months after onset) or chronic (6–9 months after onset) time points, persons randomized to early intensive motor training will show greater UE motor improvement measured at 1 year. In addition, we will use the opportunity presented to collect peripheral blood to perform a proof of principle study exploring molecular signals associated with response to treatment and overall motor recovery. See Figure 1 for a diagram of study design.

In order to adapt the rodent experimental design to the clinical delivery patterns in the United States, we made two major decisions. First was the choice of time periods in which study-related treatment would be delivered. Precisely how post-stroke days compare between humans and rodents is unknown, and we attempted to balance fidelity to the Biernaskie et al. design with the pragmatics of accommodating existing treatment venues. These venues are not under the control of the research team. Choosing the exact time points and a single day window to initiate therapies such as was used in the rodent study meant that participants would need to be consented within 72 h of stroke onset so that baseline measures could be collected and the participant randomized with the possibility of treatment beginning exactly on post-stroke Day 5. Though conceptually not impossible, this choice would lead to several complexities including unavailability of patients to undergo study related therapy during a time when diagnostic testing must take first priority, medical complications and fatigue preventing therapy participation, uncertainty about the trajectory of motor recovery, and uncertainty as to whether and where the patient might be referred for inpatient rehabilitation. We chose instead three more flexible windows of time for study related treatments: early (<30 days, corresponding to the inpatient rehabilitation period), subacute (60–90 days, corresponding to typical outpatient therapy delivery), and chronic (6 months, by which time most US stroke patients will have been discharged from therapy). These times were chosen as analogous to the 5, 14, and 30 day times used in the Biernaskie et al. study. By using those existing clinical treatment venues, any improvements in efficacy that results from this line of work can improve the effectiveness of those venues without requiring a major change in how care is delivered. Thus, the translation to actual treatment would not be hindered by the need for policy and reimbursement changes.

The second major decision was that of how much study related treatment was necessary to observe a detectable effect of a critical period. Dose-response data for motor training are particularly lacking in the first few weeks after stroke onset. We chose 20 h of additional motor therapy because our previous work has shown that a difference of 10 h of treatment is sufficient to alter motor outcomes; this amount of additional therapy should thus provide an adequate signal indicating a critical period, if there is one (Dromerick et al., 2009). Moreover, should we find a large difference in outcomes in one group, it seems feasible to deliver 20 h more training to stroke patients in the current healthcare environment. Several studies document persistent motor improvements post-stroke with treatments of similar or even less intensity (Sivenius et al., 1985; Sunderland et al., 1992; Whitall et al., 2000; Page et al., 2001; Michaelsen and Levin, 2004; Michaelsen et al., 2006; Woldag et al., 2010; Han et al., 2013).

## Methods

### Patient population

Sixty-four participants will be recruited primarily from the inpatient stroke service at MedStar National Rehabilitation Hospital (MedStar NRH). MedStar NRH hosts the Washington DC site for the NINDS StrokeNet, and recruitment from other DC area hospitals can be used if needed. Study participants will include adults with neuroimaging confirmed diagnosis of recent ischemic stroke or intraparenchymal hemorrhage. See Table 2 for a comprehensive list of inclusion/exclusion criteria.

**Table 2:** **Inclusion/exclusion criteria**.

**Inclusion criteria**
1. Ischemic or hemorrhagic stroke (with confirmatory neuroimaging) within 28 days of admission to inpatient rehabilitation.
2. Age >21 years
3. Able to participate in first study-related treatment session within 30 days of stroke onset.
4. Able to participate in all study-related activities, including 1 year follow up and blood draws.
5. Persistent hemiparesis leading to impaired upper extremity function as indicated by a score ≥1 on the NIHSS motor arm score, AND motor impairment judged clinically appropriate as defined by one or both of the following:
a. Proximal UE voluntary activity indicated by a score of ≥3 on the upper arm item of the motor assessment scale; wrist and finger movement is not required. OR
b. Manual muscle test (MMT) score of ≥2 on shoulder flexion and either elbow flexion or extension.
6. Score of ≤8 on the short blessed memory orientation and concentration scale
7. Follows 2 step commands
8. No upper extremity injury or conditions that limited use prior to the stroke
9. Pre-stroke independence: modified rankin score 0 or 1
**Exclusion criteria**
1. Inability to give informed consent
2. Prior stroke with persistent motor impairment or other disabling neurologic condition such as multiple sclerosis, parkinsonism, ALS, dementia requiring medication
3. Rapidly improving motor function
4. Clinically significant fluctuations in mental status in the 72 h prior to randomization
5. Hemispatial neglect as determined by an asymmetry >3 errors on the mesulam symbol cancellation test.
6. Not independent prior to stroke (determined by scores of <95 on barthel index or >1 on modified rankin scale
7. Dense sensory loss indicated by a score of 2 on NIHSS sensory item
8. Ataxia out of proportion to weakness in the affected arm as defined by a score ≥1 on the NIHSS limb ataxia item.
9. Active or prior psychosis within 2 years
10. Active or prior (within 2 years) substance abuse
11. Not expected to survive 1 year due to other illnesses (cardiac disease, malignancy, etc)
12. Received UE botulinum toxin within 6 months. (other meds do not exclude)

#### Study measures

The primary outcome measure, secondary outcome measures, and covariates are listed in Table 3 along with the time periods at which they will be assessed. Pre-treatment, 6 mo, and 1 yr assessments will be performed by study therapists and the assessments for the motor scales will be videotaped. A blinded therapist will view the videotapes to provide the formal assessments for each subject at the end of the study, including the ARAT primary outcome measure.

**Table 3:** **Study measures and covariates**.

**Measure**	**Domain measured**	**Baseline**	**Pretreat**	**Post-treat**	**6 Mo**	**1 Yr**
Action research arm test	Motor functional limitation (performance)	x	x	x	x	x
Motor activity log	Motor disability (self-report)	x	x	x	x	x
Nine hole peg test	Motor functional limitation	x	x	x	x	x
Functional independence measure	ADL disability	x	x	x	x	x
Motor assessment scale	UE motor functional limitation	x				
Barthel index	ADL disability	x	x	x	x	x
Fugl-meyer upper arm	Motor functional limitation (performance)	x				x
Manual muscle test	Motor strength and function	x				
Motricity index		x	x	x	x	x
Stroke impact scale-perception of change	Stroke-specific quality of life	x	x	x		x
Stroke impact scale hand-arm subscale	Stroke-specific quality of life	x	x	x		x
Modified Rankin Scale	Handicap/Global outcome	x (prestroke)	x	x		x
Activity card sort	Participation		x	x	x	x
Reintegration to normal living	Participations			x	x	x
Geriatric depression scale	Depression screen/covariate	x	x			x
NIH stroke scale	Stroke severity	x	x	x		x
Short blessed orientation memory concentration test	Dementia screen	x				
Mesulam symbol cancellation test	Visuospatial neglect	x	x	x		x
Faces scale	Pain (visual analog)	x	x	x	x	x
Medication inventory	Covariate (recovery-modifying drugs)	x	x	x	x	x
Charlson comorbidity index	Covariate (medical complexity)	x				
Oxfordshire classification	Covariate (lesion type)	x				
Edinburgh inventory	Covariate (handedness)	x				
Age	Covariate	x				

Further information on study outcome measures and covariates are provided below:

- Action Research Arm Test (ARAT): The ARAT, adapted from the Fugl-Meyer (Fugl-Meyer et al., 1975), assesses functional limitations of the UE and is the primary outcome measure for the study. It uses a 4-point ordinal scale on 19 items, where 0 indicates no movement and 3 indicates normative movement. The scale has 4 subscale scores- gross motor (9 point maximum), grasp (18 point maximum), grip (12 point maximum), and pinch (18 point maximum)- and a total scale score with a maximum of 57. The ARAT has been shown to be reliable, valid, and responsive to change across a variety of time points post-stroke (Deweert and Harrison, 1985; Hsieh et al., 1998; Van der Lee et al., 2001, 2002; Lang et al., 2006).
- Motor Activity Log (MAL): The MAL is a 28-item questionnaire measuring UE use on functional tasks (Uswatte et al., 2006; Taub et al., 2011). It has two subscales: Amount of Movement (AoM) and Quality of Movement (QoM).
- Nine Hole Peg Test: The nine hole peg test measures bimanual dexterity. The test assesses the amount of time required to position and remove nine (9) pegs on a board (Mathiowetz et al., 1992).
- Functional Independence Measure (FIM): The FIM assesses 18 item activities of daily living (Granger et al., 1993). Two subscores are created: a motor score and a cognitive score. Subscales will be analyzed separately.
- Motor Assessment Scale: The Motor Assessment Scale is a test which assesses an individual's supine to side lying, supine to sitting edge of bed, balanced sitting, sitting to standing, walking, upper-arm function, hand movements and advanced hand movements (Carr, 1992). For this study the upper-limb function portion of this test will be utilized.
- Barthel Index: The Barthel Index is the most commonly used measure of functional performance in stroke clinical trials (Shah et al., 1989).
- Upper Extremity Fugl-Meyer (UE-FM): The Fugl-Meyer will be used to assess motor function at the shoulder, elbow, wrist, and fingers (Fugl-Meyer et al., 1975). It scores reflexes and the ability to perform several simple movements and tasks on a 3-point scale. Validity and reliability of the Fugl-Meyer Test have been established (Lin et al., 2009). The Fugl-Meyer was designed for the recovery patterns observed after stroke and is most sensitive to detecting changes in subjects with moderate to severe impairment.
- Manual Muscle Test (MMT): The Manual Muscle Test evaluates strength and function of individual muscles to assess the degree of muscular weakness resulting from injury or disease (Medical Research Council, 1976). The therapist palpates each muscle while the subject voluntarily contracts the muscle against resistance. A score ranging from 0 to 5 indicates the relative strength.
- Motricity Index: The Motricity Index is a short and simple measure of motor loss primarily developed for use after stroke (Collen, 1992). This measure has proven validity and reliability and is sensitive to change in motor function after stroke.
- Stroke Impact Scale (SIS): (SIS Hand Function Subscale and SIS Recovery Scale): The SIS is a self-report scale for evaluating stroke recovery (Duncan et al., 1999). The 5-item Hand Subscale assesses hand strength, dexterity, fine, and gross motor ability associated with the ability to complete functional tasks. In the Recovery Subscale, the patient is asked to assess his or her overall degree of recovery; possible scores range from 0 to 100 with higher scores indicative of better outcomes.
- Modified Rankin Scale (mRS): The mRS measures the degree of disability and level of independence in those who suffer from neurologic injury (van Swieten et al., 1988). In addition to serving as a secondary outcome measure, the mRS will be used to gauge pre-stroke level of disability.
- Activity Card Sort (ACS): The ACS is a self-report measure of activity in four domains: instrumental activities, high and low physical-demand leisure activities, and social activities (Baum and Edwards, 2008).
- Reintegration to Normal Living: This 10 item Likert scale evaluates life satisfaction and quality of life (Stark et al., 2005).
- Geriatric Depression Scale: The 5 items version of the Geriatric Depression Scale will be used to screen for depression (Yesavage, 1988).
- NIH Stroke Scale (NIHSS): The NIHSS assesses cognitive, sensory, and motor impairments as an indicator of overall stroke severity (Brott et al., 1989).
- Short Blessed Orientation Memory Concentration Test: This test screens for dementia by assessing orientation, reasoning and short-term memory (Katzman et al., 1983).
- Mesulam Symbol Cancellation Test- This paper and pencil measure uses a cancelation task to assess visuospatial neglect (Mesulam, 1985). More than 3 omissions between the left and right visuospatial field indicates neglect.
- Pain Scale: A scale with cartoon faces that represent varying degrees of pain. The subject is asked which face reflects their current pain level (Wong et al., 2001).
- Charlson Comorbidity Index: An index that factors in a subject's medical comorbidities and age to derive a probability of 10 year survival (Charlson et al., 1987).
- Oxfordshire Classification: Classifies stroke based on neuroimaging/clinical data according to 4 possible stroke locations/severities: total anterior circulation stroke, partial anterior circulation stroke, posterior circulation stroke, and lacunar stroke (Mead et al., 2000).
- Edinburgh Inventory: A questionnaire that determines the degree to which a subject is left- or right-handed (Oldfield, 1971).

## Procedures

The trial was approved by the Institutional Review Board at MedStar National Rehabilitation Hospital/MedStar Health Research Institute (protocol #2014-065). All study subjects will provide informed consent prior to engaging in study procedures. Based on recruitment rates for the ICARE trial (Winstein et al., 2013), which was conducted at the same hospital and had somewhat similar inclusion/exclusion criteria, we anticipate all subjects for CPASS will be recruited by the end of year 2 and final outcome measures will be collected by the end of year 3.

### Randomization

The approach to randomization offers an opportunity to mitigate some of the limitations of stroke rehabilitation clinical trials. Specifically, randomization allows the investigator to decrease the impact of heterogeneity of important personal characteristics among subjects that could affect the overall study results. Ideally, all important and measurable characteristics could be balanced across the study, but there are limits to the number of variables that can be balanced, short of enrolling a sample far larger than circumstances permit. To avoid groups that are unbalanced with respect to potentially important secondary variables, we use an adaptive randomization strategy based on methods described by Atkinson (1982), Meinert (1986), and Signorini et al. (1993). The adaptive randomization scheme (Yuan et al., 2014) that we have developed increases to six the number of variables that can be balanced in this sample of 64 subjects. We have chosen to balance these variables that we have judged to be most important: age, number of days from stroke onset to baseline evaluation, ischemic vs. hemorrhagic stroke type, baseline ARAT score, concordance (dominant vs. non-dominant UE affected) and overall stroke severity as indicated by NIHSS score at baseline evaluation. We were limited to six variables to balance; we prioritized these over other potentially important variables such as the presence of a fully recovered prior stroke in the randomization. Cortical involvement was another potential randomization variable that we chose not to address because neuroimages are not always available at our center, and resources did not allow for study-related imaging. By balancing days from stroke onset to baseline evaluation, we minimize the impact of variability of time from onset to rehabilitation admission.

### Interventions

In designing the motor training intervention for CPASS, we addressed several goals for mitigating the limitations of clinical trials. These included motivating subjects as well as developing a standard treatment approach that is theory driven, measurable, and reproducible. Ideally, the treatment should be engaging and meet the clinical goals and interests of the individual participant.

One important advantage of rodent studies over human trials is the ability of the investigator to motivate maximal engagement and participation by study subjects. Rodent and human motor training paradigms are based on the work of Thorndike who proposed the “law of effect” (Thorndike, 1898). The law of effect suggests that responses closely followed by satisfaction will become firmly attached to the situation and therefore will be more likely to recur when the situation is repeated. This principle forms the basis of operant conditioning (Skinner, 1938). Operant conditioning experiments of motor training in rodents have shown that food is a powerful form of reinforcement in motor learning studies. Studies demonstrated that food pellets as positive reinforcers produced more rapid motor learning and better retention of learned responses than foot shocks as negative reinforcers in rodents (Lawson and Watson, 1963). Animals receiving highly desirable food pellets learned faster and retained more than those who were reinforced with minimally acceptable pellets. These principles form the foundation of the translation of rodent models of stroke recovery to the present treatment protocol: the use of highly salient reinforcement procedures to reinforce motor training activities.

In a human clinical trial, the use of delayed feeding and food rewards to increase motivation is not feasible. Instead, we developed an individualized treatment protocol based on the UE functional activity preferences of study participants. To standardize the elicitation of an individual participant's preferred activities, we used a widely accepted measure of leisure and functional activities. Sixty-five functional activities requiring upper extremity performance assessed by the Activity Card Sort (ACS) (Baum and Edwards, 2008) minus 24 of those activities that do not involve upper extremity involvement are presented during the treatment-planning phase in order to identify pre-stroke social, instrumental, high and low demand leisure UE activities. Study participants are asked to identify and rank the 10 most important activities that require arm and hand skills that they want to focus on during their treatment. Study participants are also able to add activities not included in the ACS. Participants rank the importance of each activity. Study therapists use these activities to develop a specific treatment plan based on a series of shaping tasks that are described below. These tasks are used for the treatment of all participants. Thus, there is customization for the goals and priorities of individual participants, but uniformity of actual motor training using the library of shaping tasks. All study participants will receive their usual and customary care (UCC) treatment as prescribed by the clinical team and approved by their insurance company. Data are collected on the number and type of UCC therapy sessions, but no attempt will be made to standardize UCC. Study related therapies will be delivered by research-employed certified physical or occupational therapists; the study team meets twice weekly to review treatment related issues and insure reproducibility.

Persons randomized to the early group (<30 days) have study-related treatments started as quickly as possible; those randomized to a later time point will have their treatment started as close as possible to the first day of their treatment window (2 mo. or 6 mo. post-stroke for the subacute and chronic groups respectively). By concentrating therapy for the early group as much as feasible, overlap with the subacute group will be minimized or eliminated. Study related treatments will be delivered in addition to UCC, and will consist of 20 h of therapy delivered in 10 two-hour sessions 2-3x/week. In the United States, stroke patients admitted to the acute inpatient rehabilitation setting are generally required to receive 3 h of therapy daily; however a multisite study (Lang et al., 2009) demonstrated a paucity of time devoted to restorative motor training that was remarkably consistent at less than 10 min daily. Thus, the additional study-related training overwhelms any differences in the amount of UCC training delivered to individual study participants. To avoid reproducing the High Intensity condition used in the VECTORS trial, individuals randomized to the early/acute group will only receive 1 additional hour of treatment per session until discharge from in-patient rehab, and then will receive 2 h of treatment per session similar to the subacute and chronic groups. This training will focus exclusively on UE motor training, and the use of the more affected UE in the activities identified by the baseline. This treatment will be delivered in the inpatient rehab setting (for those randomized to the early group) or outpatient clinic settings (for subacute and chronic groups) whenever possible. If needed, the study therapy will be provided in the home to achieve the requisite 20 h of treatment.

Balancing the need to customize study-related treatment to the goals and preferences of individual participants (thereby maximizing motivation) while at the same time delivering a measurable, reproducible, and theory driven motor training program is important. The shaping treatment developed for use in VECTORS, an NINDS-funded Phase II trial (Dromerick et al., 2009) will be included in the 20 h of therapy. Constraint will not be used. Shaping is a process used in both human and rodent motor training after stroke. There are many theories of why shaping produces lasting changes in motor performance (Taub et al., 1994; Ingvaldsen, 1997; Winstein et al., 2013). The selection of tasks is very important since the participant must be committed to achieving the goal despite the difficulty of task performance. The therapist grades the tasks to provide enough challenge to encourage progress while guarding against frustration and failure which act as negative reinforcers and decrease the probability of full participation in the treatment program. The therapist offers positive reinforcement in the forms of verbal and visual feedback. Task difficulty is increased when the participant completes two sets of 10 correct repetitions of task performance on two consecutive days of treatment.

### Statistical analysis

Power analysis estimated that a sample size of 64 (16 per study arm) is sufficient to test the study hypotheses based on the standard deviations and treatment differences observed in the VECTORS trial. The study sample size is determined based on demonstrating a moderate effect size (0.425 as observed in the preliminary data) in the primary endpoint, the ARAT at 1 year in an ANOVA model, with 80% power at a significance level of 5%. The specific comparison of the treatment at each time point will be conducted using contrasts. One interim analysis is planned when the number of patients with 1 year ARAT reaches 32. This analysis following the adaptive group sequential procedure allows us the opportunity to spot treatment time points with pronounced improvement in ARAT or the lack thereof while maintaining the rigor (e.g., Type I error) of the trial.

The general linear model (ANOVA) will be used for group comparisons on the primary endpoint ARAT using intent to treat principle. All subjects recruited into the study will be included in this primary analysis except the run-in participants. Additional per protocol secondary analyses will be performed. The timing of treatment hypothesis will be addressed by contrasts between the four treatment groups at 1 year after stroke. The magnitude of these effects will be computed. The analysis on secondary endpoints will be mostly descriptive in nature given the limited sample size in this pilot trial.

We will also examine additional covariates to ascertain which, if any, of these covariates are correlated with treatment response, we will carefully examine the data for indications of interactions between covariates and treatment effects. Cross validation for the selected covariates predictive of patient outcome will be performed. Such covariates will then be considered as potential exclusion criteria for future trials of therapy dosing during identified sensitive periods after stroke.

Analysis of the motor function measures and their correlation with clinical scales of UE motor function will allow us to select the particular parameters to be retained in future trials should a sensitive period be found. If the effects are large, a subsequent trial can be designed in a more economical fashion.

Data obtained from the skill and activity rankings will be analyzed using Spearman rank coefficients. Skill preference scores per category will be calculated by summing the total preference scores belonging to a specific skill category. Overall satisfaction scores will be computed for the selected skills following the standard scoring procedures established for the Motor Activity Log. These summed scores will be analyzed using appropriate parametric or non-parametric repeated measures analyses based on the distribution of the scores. The correlative analysis with multi-omic assessments is more descriptive given the limited sample size and is described further in the next section.

### Peripheral blood draws for multi-omic assessments

The CPASS trial also presents an opportunity to determine whether the same molecular processes that occur in experimental animals post-stroke also take place in the human. The last decade has produced an explosion in the analytic methods used to assess various clinical specimens. Blood biomarker analysis in human stroke is not new, but most of the focus up to this point has remained in the hyperacute phase—finding biomarkers to diagnose acute stroke (Tang et al., 2006). Blood biomarker analysis is viable because many brain-derived molecules cross the blood-brain-barrier, including micro-RNA's, lipids, short peptide chains, and exosomes. Based on similar analyses from relevant conditions such as TBI (Diaz-Arrastia et al., 2014) and Alzheimer's Disease (Mapstone et al., 2014), we believe there is a reasonable expectation of useful molecular signals. Additionally, it seems possible that peripheral metabolism might also affect recovery.

In order to limit sample variability in the CPASS trial, whenever possible, blood draws will be performed on fully awake subjects, between 8 a.m. and 10 a.m., who are fasting at least since midnight, and have not yet taken their morning medications. Multi-omic analyses requires collection of blood into three lavender top (EDTA) tubes (7 ml) and three PAXgene tubes (2.5 ml), which are processed and stored for later analysis at study conclusion. To minimize costs and subject burden, we will limit blood draws to baseline, pre-treatment, and post-treatment assessments.

### Multi-omics analysis from peripheral blood specimens

We plan to use a variety of methods in analyzing human blood specimens from subjects in the CPASS trial. In this article we will use the term multi-omics to represent what some call panomics or “pan-omics,” terms used to identify a group of molecular biological methods and technologies, including metabolomics, proteomics, transcriptomics, epigenomics, genomics, and exosomics. While each of these methods has the potential to provide important insights, integration of their combined analytic results may further elucidate the biological basis of human stroke recovery.

To our knowledge, there are no completed studies testing multi-omics approaches during the recovery phase of human stroke. We know of two studies currently underway: the START trial (Carey et al., 2013), which seeks to identify a gene profile for post-stroke depression; and BAPTISe (Nave et al., 2013), which looks for changes in specific lipids and proteins related to post-stroke cardiovascular training. Our approach to molecular analysis in CPASS differs in many ways. In CPASS, we seek to identify profiles of differentially expressed genes, lipids, and proteins in the baseline blood draw that will distinguish those with good recovery from those with poor recovery at 1 year. In addition, we will compare blood samples drawn immediately before and after the 20 h of additional OT in the subacute and chronic groups with the hope of identifying molecular changes associated with OT. Given the small number of subjects in each group (16), omics findings for therapy-related changes will largely be descriptive in nature. Similar to START, but unlike BAPTISe, we will take an untargeted approach to multi-omics. In other words, we will not identify particular genes, lipids, and proteins *a priori*, but rather will use advanced bioinformatic techniques to identify candidate biomarkers. Our past experience suggests this untargeted approach is an effective method to identify novel biomarkers in neurologic disease (Mapstone et al., 2014). Another important way in which our analysis differs is that it will include exosomics. Exosomes are small vesicles released by almost all cell types including neurons. Central nervous system (CNS) exosomes appear to exit to the periphery and comprise at least 10–20% of plasma exosomes (Fiandaca et al., 2014). We anticipate that the analysis of protein, lipid, and RNA cargos from the CNS-derived exosomes in plasma may allow us to differentiate biological changes taking place in the CNS from those occurring in the periphery during stroke recovery.

## Discussion: challenges of translating an animal experimental paradigm into a human clinical trial

Laboratory experiments using rodents have many advantages that allow the most exacting study of the biology of mammalian brain recovery, and the most unequivocal demonstration of the impact of putative motor training interventions. At the same time, the application of the scientific findings uncovered in the laboratory is motivated in large part by improving brain injury recovery in humans. For the translation of rodent findings to human disease, demonstration that the findings in rodents generalize to humans is essential, and requires clinical trials. We have designed a human stroke motor recovery trial tightly linked to the methods used in rodent motor recovery experiments done in the laboratory. Our design decisions have been made with the goals of minimizing the real-world limitations of rehabilitation trials and maximizing the clinical and scientific utility of our efforts and those of our study participants. A single center trial allows for maximal control of experimental conditions, but that control cannot approach that of a rodent laboratory.

The CPASS trial lays the groundwork for determining whether critical periods truly exist following human stroke. This phase II trial will provide the necessary data to conceive a well-planned definitive multicenter trial. The CPASS trial will navigate the challenges of delivering study-based occupational therapy in three different settings: during inpatient rehabilitation, outpatient rehabilitation, and in the patient's home. By measuring the primary endpoint at 1 year, we ensure that any treatment related difference between groups is durable—a key feature if we are to prove the existence of a critical period. Elucidation of molecular markers for recovery might lead to lab tests that could identify sensitive periods, when persons with stroke can best use treatment resources to achieve their goals. This could also someday lead to drug development that could prolong or restore sensitive periods during stroke recovery, giving persons with stroke a better recovery and thus more opportunity to choose goals and more fully participate in society. We hope that the advances explored by CPASS will lead to new discoveries in the field and ultimately better outcomes for stroke patients.

## Author contributions

All authors participated in experimental design and preparation of the manuscript. DE, MG, JB, KB, and EC were primarily responsible for Sections Study Measures and Interventions. MT was primarily responsible for Sections Randomization and Statistical Analysis. Members of the Biomarker Laboratory and Biorepository at Georgetown University Medical Center (IT, RC, MO, RP, AC, MM, MF, and HF) were primarily responsible for Sections Peripheral Blood Draws for Multi-omics Analysis and Multi-omics Analysis from Peripheral Blood Specimens.

### Conflict of interest statement

Drs. Federoff, Fiandaca, Cheema, and Mapstone are named as co-inventors on provisional patent applications that have been filed by Georgetown University and the University of Rochester related to specific biomarker technologies described in this article. The authors declare that the research was conducted in the absence of any commercial or financial relationships that could be construed as a potential conflict of interest.
